# Reducing AD-Like Pathology in 3xTg-AD Mouse Model by DNA Epitope Vaccine — A Novel Immunotherapeutic Strategy

**DOI:** 10.1371/journal.pone.0002124

**Published:** 2008-05-07

**Authors:** Nina Movsesyan, Anahit Ghochikyan, Mikayel Mkrtichyan, Irina Petrushina, Hayk Davtyan, Purevdorj B. Olkhanud, Elizabeth Head, Arya Biragyn, David H. Cribbs, Michael G. Agadjanyan

**Affiliations:** 1 Department of Molecular Immunology, Institute for Molecular Medicine, Huntington Beach, California, United States of America; 2 Institute for Brain Aging and Dementia, University of California Irvine, Irvine, California, United States of America; 3 Immunotherapeutics Unit, Laboratory of Immunology, National Institute on Aging, Baltimore, Maryland, United States of America; 4 Department of Neurology, University of California Irvine, Irvine, California, United States of America; Massachusetts General Hospital and Harvard Medical School, United States of America

## Abstract

**Background:**

The development of a safe and effective AD vaccine requires a delicate balance between providing an adequate anti-Aβ antibody response sufficient to provide therapeutic benefit, while eliminating an adverse T cell-mediated proinflammatory autoimmune response. To achieve this goal we have designed a prototype chemokine-based DNA epitope vaccine expressing a fusion protein that consists of 3 copies of the self-B cell epitope of Aβ_42_ (Aβ_1–11_) , a non-self T helper cell epitope (PADRE), and macrophage-derived chemokine (MDC/CCL22) as a molecular adjuvant to promote a strong anti-inflammatory Th2 phenotype.

**Methods and Findings:**

We generated pMDC-3Aβ_1–11_-PADRE construct and immunized 3xTg-AD mouse model starting at age of 3–4 months old. We demonstrated that prophylactic immunizations with the DNA epitope vaccine generated a robust Th2 immune response that induced high titers of anti-Aβ antibody, which in turn inhibited accumulation of Aβ pathology in the brains of older mice. Importantly, vaccination reduced glial activation and prevented the development of behavioral deficits in aged animals without increasing the incidence of microhemorrhages.

**Conclusions:**

Data from this transitional pre-clinical study suggest that our DNA epitope vaccine could be used as a safe and effective strategy for AD therapy. Future safety and immunology studies in large animals with the goal to achieve effective humoral immunity without adverse effects should help to translate this study to human clinical trials.

## Introduction

A critical aim in developing therapeutic interventions for Alzheimer's Disease (AD) is the identification of suitable targets. It is estimated that there are currently about 18 million people worldwide with AD. This number is projected to nearly double by 2025 to 34 million (http://www.who.int). Current data suggest that the accumulation of neurotoxic amyloid-β-(Aβ) may play a central role in the onset and progression of AD [1]. Accordingly, many therapies for AD are aimed at reduction of the level of Aβ in the brain and/or blocking assembly of the peptide into pathological forms that disrupt cognitive function [2]. A potentially powerful strategy is immunotherapy with anti-Aβ antibody that could facilitate the reduction pathological forms of Aβ in the brain. Pre-clinical and clinical trials have demonstrated that both active [3]–[6] and passive [7] vaccination strategies have been effective in mice and may be effective in patients with AD [8]–[10]. However, the first immunotherapy clinical trial in AD patients was halted when a subset of individuals immunized with the vaccine containing Aβ_42_ formulated in a Th1-type adjuvant developed adverse events in the central nervous system [8]–[15]. While the actual cause of the adverse events is unknown, speculation has centered on autoreactive cellular immune responses specific for the T-cell epitope in Aβ, or possibly the adjuvant, and/or the reformulation of the vaccine during the Phase IIa study [9], [10], [12], [13], [16]. In an attempt to avoid the problems associated with active immunization of elderly AD patients other strategies have been proposed [17], [18]. One of them, passive transfer of therapeutically potent humanized monoclonal anti-Aβ antibody to AD patients, is currently in clinical trial (AAB-001).

Another promising strategy is active vaccination with an epitope vaccine composed of the immunodominant self B cell epitope of Aβ_42_ and a non-self T helper (Th) cell epitope/s. We recently demonstrated the feasibility of this strategy in wild-type [19] and APP/Tg mice [20], [21]. Data with a peptide epitope vaccine composed of Aβ_1–15_ or Aβ_1–11_ and a non-self promiscuous T cell epitope, PADRE [22] suggest that this active immunotherapy strategy could be effective and safe in humans, because this vaccine (i) should bind to almost all human HLA-DR molecules with high affinity; (ii) does not induce autoreactive Th cells while generating strong non-self Th responses; and (iii) induces production of high titers of anti-Aβ antibody. Interestingly, Elan and Wyeth used a similar strategy for the development of an AD protein vaccine (ACC-001), in which an N-terminal sequence of Aβ is conjugated to diphtheria toxin that should provide foreign Th epitope/s. However, some problems associated with the development of safe and effective epitope vaccines may still exist. For example, a peptide epitope vaccine is immunogenic when it is synthesized on a multiple antigenic peptide (MAP) backbone, but cannot be easily scaled up for clinical studies. Another issue associated with a peptide epitope vaccine strategy is the requirement for a potent conventional adjuvant. The only adjuvant currently approved for use in humans is Alum, which may not be capable of inducing strong anti-Aβ immunity in elderly patients as was previously shown in mice [23].

To avoid these problems, we have generated a novel vaccine by combining our epitope vaccine approach to a DNA vaccine strategy. Previously, our group [17], [24] and later other authors [25]–[28] generated a DNA vaccine based on full-length Aβ_42_. Unfortunately, all of these vaccines induced, at best, only low titers of anti-Aβ_42_ antibody in APP/Tg mice. In addition, this type of vaccine should not be considered safer than fibrillar Aβ_42_ used in AN-1792 trial, because it possesses both self-B and self-T cell epitopes within the Aβ peptide. The goal of this study was to develop a fundamentally different and safer DNA vaccine that could induce strong anti-Aβ antibody without generation of potentially harmful autoreactive T cells. Accordingly, we prepared a plasmid encoding an immunodominant self-B cell epitope of Aβ_42_, a foreign promiscuous T cell epitope, PADRE along with a molecular adjuvant, macrophage-derived chemokine (MDC/CCL22), and tested the efficacy of this vaccine in a 3xTg-AD mouse model. We hypothesized that this vaccine should induce strong anti-Aβ antibody responses with help from the MDC-driven anti-inflammatory (Th2-type) CD4^+^Th cells specific to non-self antigen, PADRE.

## Results

### DNA epitope vaccine induces strong Aβ-specific antibody production using help from PADRE-, but not Aβ-specific Th cells

**Figure 1:**
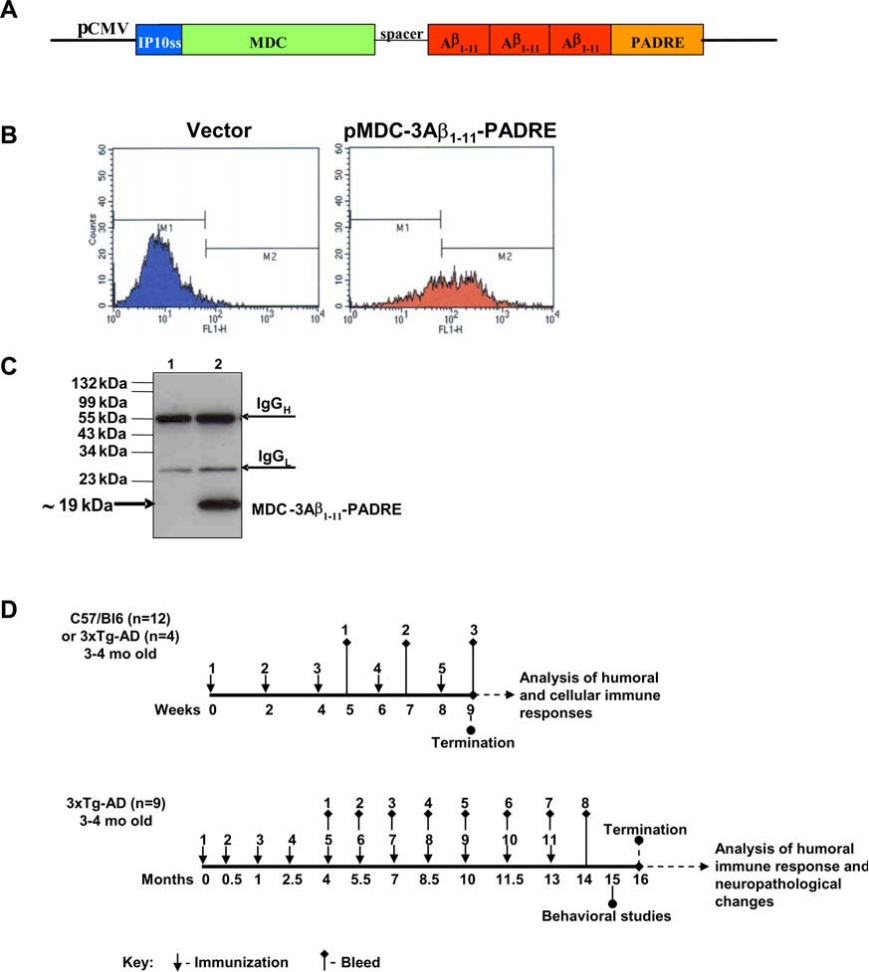
Schematic representation of DNA vaccine construct, its expression, and immunizations schedules. A) Fusion gene encoding epitope vaccine, 3Aβ_1–11_ and PADRE (aKXVAAWTLKAAaZC, x = L-cyclohexylalanine, Z = aminocaproic acid) was introduced into pCMVE-AB/hMDC in frame to the 3′-end of mature *MDC* exactly as shown. *MDC* gene is fused to the IP10 signal sequence in its 5′-end and to the *3Aβ_1–11_-PADRE* minigene in its 3′-end through small spacer. Transcription of *MDC-3Aβ_1–11_-PADRE* gene is controlled by CMV promoter/enhancer. B) To analyze the intracellular expression of pMDC-3Aβ_1–11_-PADRE, transiently transfected CHO cells were permeabilized/fixed and stained with anti-Aβ 6E10 antibody followed by FITC-conjugated anti mouse IgG. Cells were analyzed by FACScan. Approximately ∼65% of CHO cells transfected with pMDC-3Aβ_1–11_-PADRE showed 6E10-positive staining. Cells transfected with control vector showed only background staining. C) The secretion of MDC-3Aβ_1–11_-PADRE by transfected CHO cells was demonstrated by IP/WB. MDC-3Aβ_1–11_-PADRE protein was immunoprecipitated from the growth medium of transfected CHO cells with anti-Aβ antibody (6E10), separated by 15% Tris-SDS PAGE and transferred onto nitrocellulose membrane. Proteins were visualized by staining with 6E10 followed by HRP-conjugated anti-mouse IgG. Lane 1 represent vector (pCMVE-AB/MDC); Lane 2 represent Epitope Vaccine (pMDC-3Aβ_1–11_-PADRE). D) 3–4 mo old C57/Bl6 (n = 6) or 3xTg-AD (n = 4) mice were immunized 5 times biweekly. Blood was collected after 3^rd^, 4^th^ and 5^th^ immunizations and humoral immune response was analyzed in sera. Seven days after the last immunization mice were sacrificed and cellular immune response was analyzed in splenocytes. Experiment with C57/Bl6 mice was repeated (total n = 12). Next, 3–4 mo old 3xTg-AD (n = 9) mice were immunized 11 times as indicated. Blood was collected and humoral immune response was analyzed in sera. After 11^th^ immunization behavioral studies were conducted and upon its completion, mice were sacrificed and neuropathological changes were analyzed in the brains of experimental and control mice.

Previously, we have utilized MDC/CCL22 to induce a Th2-type immune response to DNA vaccine encoding tumor or viral antigens and generated high titers of therapeutic antibody [29], [30], suggesting that this may also enable us to develop a safe and potent AD vaccine. To test this hypothesis, we have engineered a DNA vaccine construct that encodes MDC fused in frame with three copies of the immunodominant self-B cell epitope of Aβ_42_ (3Aβ_1–11_), and foreign Th cell epitope, PADRE [22] (pMDC-3Aβ_1–11_-PADRE, Figure 1A). The construct expressed and secreted MDC-3Aβ_1–11_-PADRE fusion protein (∼19 kDA), as shown by intracellular staining of pMDC-3Aβ_1–11_-PADRE transfected CHO cells (Figure 1B) and by immunoprecipitation of the conditioned media (Figure 1C).

**Figure 2:**
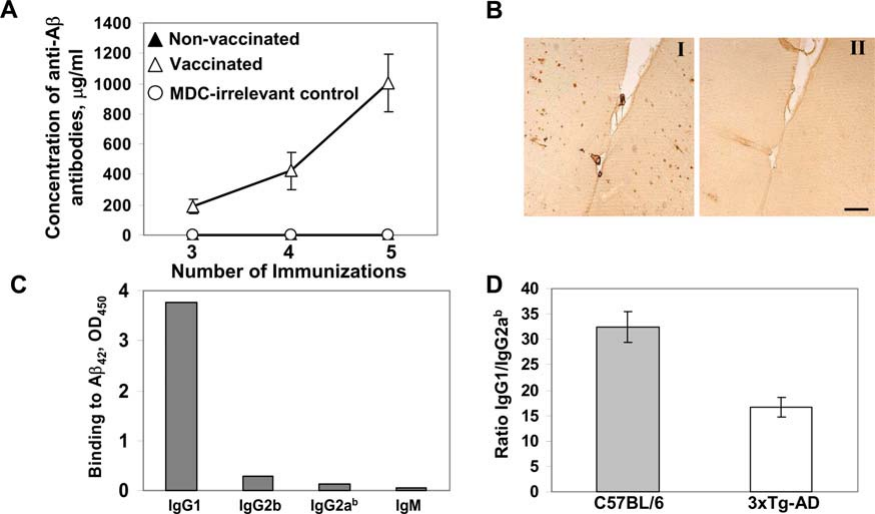
DNA epitope vaccine induces strong Th2 polarized humoral immune responses specific to human Aβ. A) DNA epitope vaccine induces very strong humoral immune response in 3–4 mo old C57/Bl6 mice (n = 6). Group of non-vaccinated (n = 6) as well as group of mice (n = 6) injected with plasmid encoding MDC fused with irrelevant antigen did not generate anti-Aβ antibody. B) Anti-Aβ antibody generated in mice immunized with DNA epitope vaccine recognizes human Aβ deposits when used in immunohistochemical experiments in an AD case, and this binding is blocked by pre-absorption of sera with both Aβ_42_ or Aβ_1–11_ peptide. C) DNA epitope vaccine induces the production of anti-Aβ antibody of predominantly IgG1 isotype in wild-type (C57/Bl6) animals. D) IgG1/IgG2a^b^ ratio in wild-type (C57/Bl6) and 3xTg-AD mice was equal to 32.4±3.2 and 16.7±2, respectively, which is an indirect measurement of Th2-type immune response induced by DNA epitope vaccine. Of note, all experiments with C57Bl/6 mice were repeated (n = 6) with the similar results.

**Figure 3:**
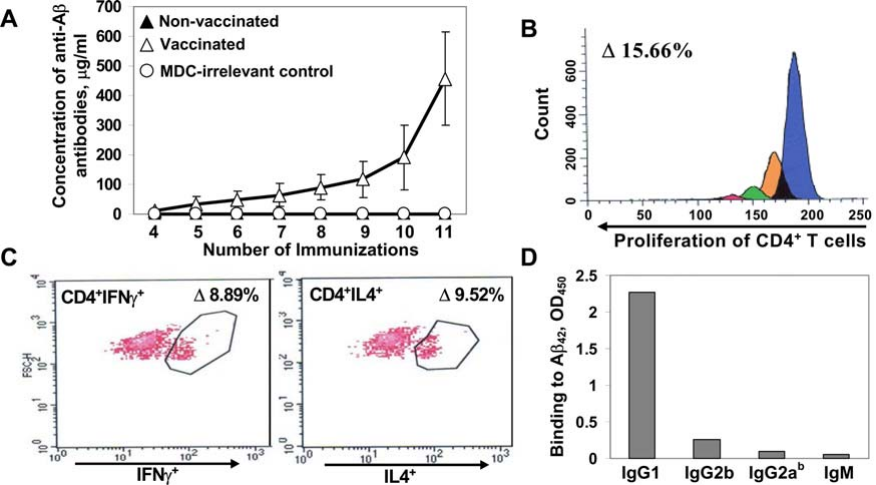
DNA epitope vaccine induces production of high concentrations of anti-Aβ antibody in 3xTg-AD mice and activates proliferation of Th2-polarized PADRE-specific CD4^+^T cells. A) Concentrations of anti-Aβ antibody were detected in the sera of individual animals (n = 9 vaccinated mice, n = 5 MDC-irrelevant control, n = 5 non-vaccinated mice) after each immunization. B–C) Proliferation of CD4^+^T cells (B) and production of cytokines by this T cell subset (C) were detected by flow cytometry in pooled splenocyte cultures (n = 4) after 5^th^ immunization. Data are presented after subtraction of the percent of proliferating or cytokine-producing CD4^+^ T cells in non-re-stimulated immune splenocyte cultures from the percent of proliferating or cytokine-producing cells detected in the cultures of splenocytes re-stimulated with PADRE. D) Th2-polarization was confirmed by antibody isotypes analysis: vaccine induced primarily IgG1 type antibody.

To test the immunogenicity of our DNA epitope vaccine *in vivo*, both 3xTg-AD and C57/Bl6 mice of H2^b^ immune haplotype were immunized with pMDC-3Aβ_1–11_-PADRE (Figure 1D), and cellular and humoral immune responses were assessed. Impressively, we detected strong humoral immune responses in both strains of vaccinated mice (Figures 2A and 3A) as we previously reported with peptide epitope vaccine formulated in a strong Th1-type conventional adjuvant [20], [21]. In contrast, wild-type and 3xTg-AD mice immunized with control MDC construct fused with an irrelevant tumor antigen [30] did not produce anti-Aβ antibody (Figure 2A, 3A). Of note, although 3xTg-AD mice responded to the DNA vaccination by production of high titers of anti-Aβ antibody (456.3±157 µg/ml) this response was significantly lower than in wild-type mice in accordance with published results with Aβ peptide vaccines [31], [32]. Assuming that there is a possibility of some antibody being already bound up with the Aβ peptide in plasma of 3xTg-AD, but not wild-type mice, thus lowering the apparent titers, we analyzed anti-Aβ antibody concentrations in immune sera after dissociation of antibody-antigen complexes as described by [33]. It is appeared that dissociation of antigen-antibody complexes did not increase the titers of anti-Aß antibody in sera collected from immune 3xTg-AD mice (Figures 2A and 3A). In other words, the difference in concentrations of antibody detected after immunizations of wild-type and 3xTg-AD mice cannot be explained by binding of antibody to circulating plasma Aβ molecules. It is possible that the mechanism of B-cell tolerance is involved in lowering titers of anti-Aβ antibody in vaccinated 3xTg-AD mice [34].

Next, we analyzed therapeutic potency of anti-Aβ antibody from immune 3xTg-AD mice *in vitro*. As we expected from our previous studies, this antibody bound not only to Aβ_42_ peptide by ELISA, but also to monomeric, oligomeric, and fibrillar forms of this human peptide in dot blot assay (data not shown), and Aβ deposits in brain tissue from an AD case (Figure 2B).

As we expected, the DNA epitope vaccine also induced robust anti-PADRE, but not anti-Aβ T cell immunity in 3xTg-AD mouse model. More specifically, DNA vaccine activated CD4^+^T cells after their *in vitro* re-stimulation with PADRE (Figure 3B), but not Aβ_40_ peptide (data not shown). This anti-PADRE cellular immune response that ultimately provides help to Aβ-specific B cells was Th2-polarized, as was shown by FACS assay used for the detection of Th1- (IFNγ) or Th2- (IL-4) type intracellular cytokines (Figure 3C). These data on generation of PADRE, but not anti-Aβ specific Th cells were confirmed in two independent experiments with vaccinations of C57/Bl6 mice (data not shown) and supported our previous results generated with peptide epitope vaccine [20], [21]. In addition, the subclass of IgG that is induced after immunization is an indirect measure of the relative contribution of Th2-type cytokines versus Th1-type cytokines [35]. As shown in Figure 3D, immunization of 3xTg-AD mice with the DNA epitope vaccine induced primarily a Th2-biased humoral immune response. Such IgG1-polarized antibody responses were also detected in wild-type mice vaccinated with the DNA epitope vaccine (Figure 2C). The IgG1/IgG2a^b^ ratios in the immunized non-transgenic and 3xTg-AD mice were equal to 32.4±3.2 and 16.7±2, respectively (Figure 2D). Thus, our construct with the MDC molecular adjuvant generates a high concentration of Aβ-specific antibody and directs immune responses in both wild-type and 3xTg-AD mice towards an anti-inflammatory Th2-phenotype.

### DNA epitope vaccine prevents cognitive dysfunction in immunized animals

**Figure 4:**
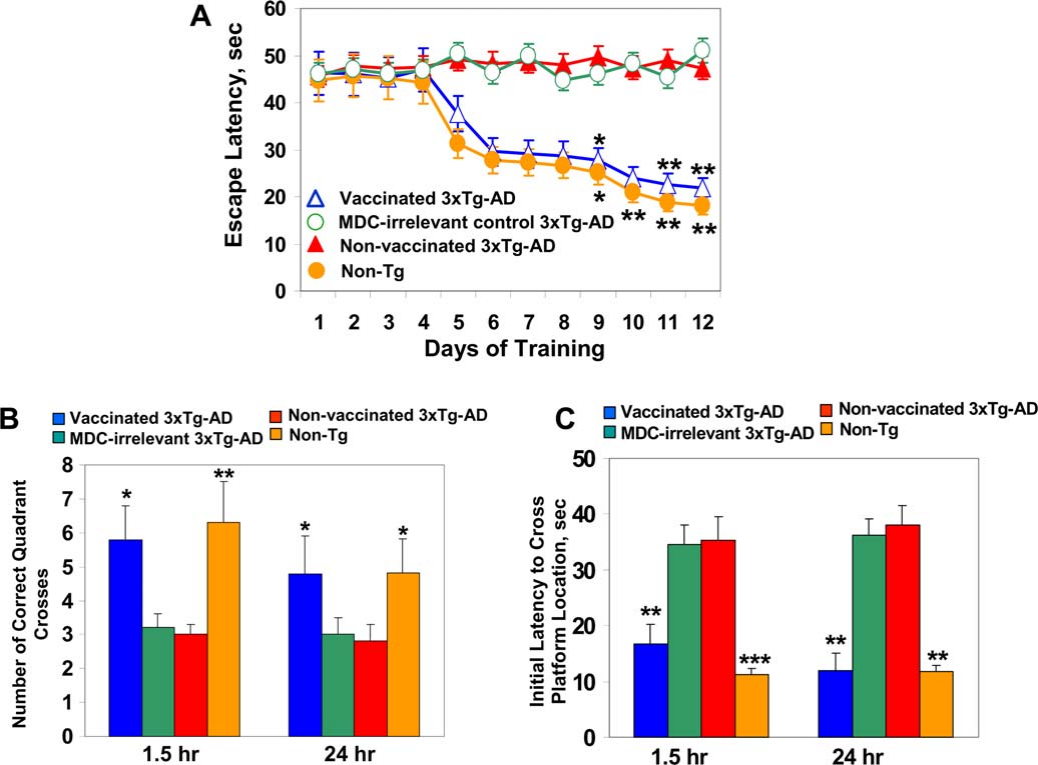
DNA epitope vaccine improves acquisition and retention of spatial memory in immune mice. A) The escape latency to reach the platform was significantly decreased in the groups of non-transgenic (n = 6) and 3xTg-AD immune mice (n = 9) versus age-matched groups of MDC-irrelevant control (n = 5) and non-vaccinated control 3xTg-AD mice (n = 5). Four trials per training day for each mouse were used until the criterion was reached in average ≤20 sec; maximal time allowed to find the platform = 60 sec. B) The number of correct quadrant crosses was significantly higher in a group of immune 3xTg-AD mice versus age-matched control groups for both 1.5- and 24-hr (short and long memory, respectively) probe trials. Of note, we did not find significant differences for the time spent in the quadrant opposite to the quadrant containing the platform during the training (data not shown) C) In both 1.5- and 24-hr probe trials the initial latency to cross the platform location was significantly lower in a group of immune mice versus both groups of age-matched controls. *P<0.05; **P<0.01; ***P<0.001 denote significant differences with respect to both MDC-irrelevant control and non-vaccinated 3xTg-AD mice of the same age. Error bars indicate SD.

To demonstrate that vaccination initiated in young 3xTg-AD mice without pre-existing AD-like pathology is beneficial as they age to 18±0.5 mo old, we measured spatial learning and memory in control (naïve or immunized with plasmid encoding MDC fused with irrelevant antigen) and immunized animals using the Morris Water Maze (MWM) [36]. Additionally, a fourth group consisting of age-matched non-transgenic mice of the same haplotype was used as a pathology-free control. As shown in Figure 4A, the latency to reach the platform for 3xTg-AD immunized mice decreased on day 5 of training and was significantly lower for days 9-10 and 11-12 (P<0.05 and P<0.01, respectively) compared to the groups of control 3xTg-AD mice. Notably, this latency decrease was similar to a group of non-transgenic mice of the same age. Retention (probe trials) for spatial information was assessed 1.5 and 24 hours after the last training trial. In the 1.5-hour probe trial the number of correct quadrant crosses was significantly higher (P<0.05) in immunized 3xTg-AD mice compared to the 3xTg-AD controls. In the 24-hour probe trial spatial memory was also significantly improved in the experimental group (P<0.05) compared to both control groups and was similar to non-transgenic mice (Figure 4B). Statistically significant improvements (P<0.01) were also observed when analyzing the initial latency to reach the platform for immunized 3xTg-AD mice compared to the control transgenic mice for both 1.5- and 24-hour probe trials (Figure 4C). These data illustrate that vaccinated mice retain spatial information as well as non-transgenic mice, and significantly better than non-vaccinated 3xTg-AD mice do, i.e. both short-term (1.5 hr) and long-term (24 hr) spatial reference memory were improved in immunized animals. Thus, DNA epitope vaccine, but not plasmid, encoding MDC fused irrelevant antigen prevented age-related cognitive decline in 3xTg-AD mice.

### DNA epitope vaccine prevents development of AD-like pathology in immune animals

**Figure 5:**
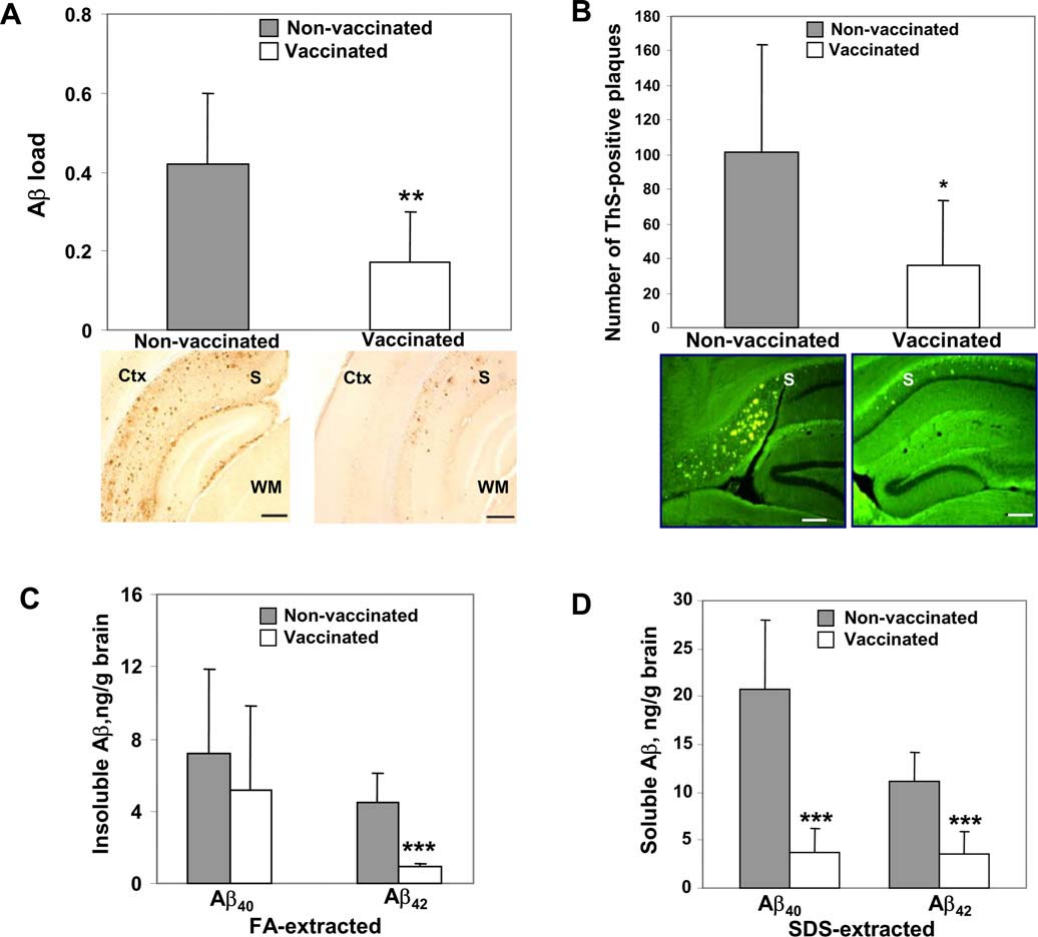
DNA epitope vaccine reduces Aβ depositions in the brains of immune 3xTg-AD mice at age 18±0.5 mo. A) A significant reduction in Aβ load (6E10-immunoreactive core and diffuse plaques) was detected in the hemibrains of vaccinated mice compared to the control animals (**P<0.01). Representative images of immunized and control mice hemibrains stained with 6E10 (scale bar 50 µm), Ctx-cortex, S-subiculum, WM-white matter. B) A significant reduction of ThS-positive core Aβ plaques was observed in the hemibrains of vaccinated mice (*P<0.05) compared to the control animals. Shown are representative images of ThS-stained hemibrains of vaccinated or control mice were (scale bar 300 µm), S-subiculum. C) As a result of DNA epitope vaccination, significant reduction (***P<0.001) of insoluble Aβ_42_ level in brain homogenates was observed, although, decrease in level of insoluble Aβ_40_ was not statistically significant. D) Both soluble Aβ_40_ and Aβ_42_ levels in brain homogenates of immunized mice were significantly reduced (***P<0.001) following epitope vaccine immunization. Bars represent average±SD for n = 5 in the group of control mice, and n = 9 in the group of vaccinated 3xTg-AD mice.

**Figure 6:**
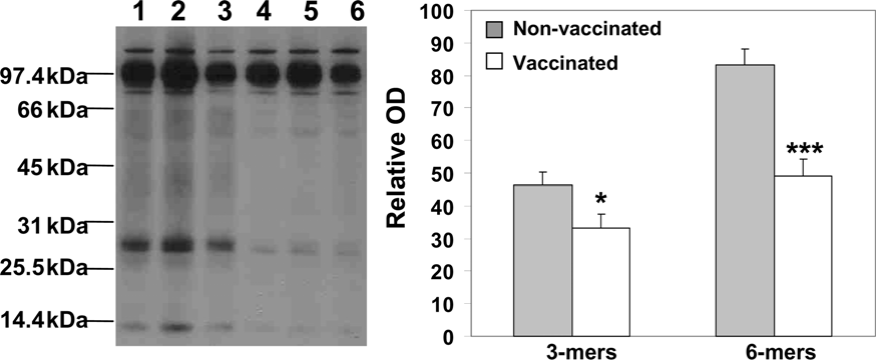
The level of oligomeric forms of Aβ detected in hemibrain homogenates by combination of IP with WB using biotinylated anti-Aβ 20.1 monoclonal antibody. Densitometric quantification of bands (relative optical density) revealed significant reduction in the level of Aβ oligomers (3-mers and 6-mers) in brain extracts from immune mice in comparison to control animals (*P<0.05 and ***P<0.001, respectively). Of note, in these experiments we did not detect clear bands of higher molecular weight oligomers in the soluble extracts of proteins from brains of vaccinated or non-vaccinated 3xTg-AD mice. Representative picture of WB is presented (Lanes 1-3 non-vaccinated, lanes 4-6 vaccinated).

Vaccination significantly reduced Aβ burden (both diffuse and core plaques) in several areas of the brains of immune mice versus control animals at age 18±0.5 mo (Figure 5A). These data were confirmed by analysis of Thioflavin S-positive core plaques measured in the brains of experimental and control mice (Figure 5B). Collectively, these results suggest that the DNA epitope vaccine is effective in reducing amyloid burden (diffuse and core plaques) in the brains of 3xTg-AD mice, which at the start of immunization did not possess Aβ pathology (protective vaccination). A potential problem of immunotherapy could be the fact that a reduction of insoluble Aβ may lead to increased levels of soluble forms of this peptide [14], primarily oligomers, the most toxic form for neurons, and impair cognitive function [2]. As demonstrated in Figure 5C DNA epitope vaccination induced a significant reduction (P<0.001) of insoluble Aβ_42_, but not Aβ_40_ peptides in the brains of immunized mice compared to control animals. Importantly, we observed that vaccination significantly reduced (P<0.001) the levels of potentially toxic forms of amyloid, soluble Aβ_42_ and Aβ_40_ peptides in the brains of immunized 3xTg-AD mice (Figure 5D). We further confirmed these results after comparing experimental and control brain homogenates by a combination of immunoprecipitation and Western blotting. Our data demonstrate that 20.1 monoclonal antibody (MoAb)-reactive Aβ oligomers, primarily 3- and 6-mers (∼14 and ∼28 kDa) were significantly reduced in the brains of immunized mice (Figure 6).

**Figure 7:**
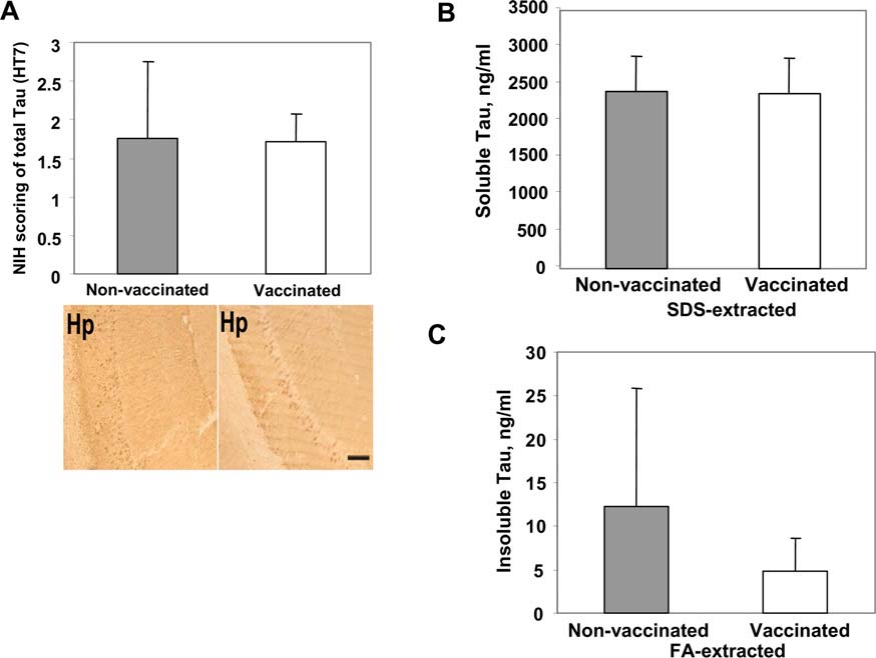
DNA epitope vaccine does not affect the level of soluble and insoluble tau in the brains of immune mice. A) Image analysis of immunohistochemical staining showing no differences in the levels of total tau (HT-7) in the hemibrains of vaccinated and control mice (scale bar 50 µm), Hp-hippocampus. B–C) Quantitative analysis (ELISA) of SDS-extracted (soluble) tau level revealed no differences between vaccinated and control mice (B). Decrease in the levels of FA-extracted (insoluble) tau was not statistically significant (C).

**Figure 8:**
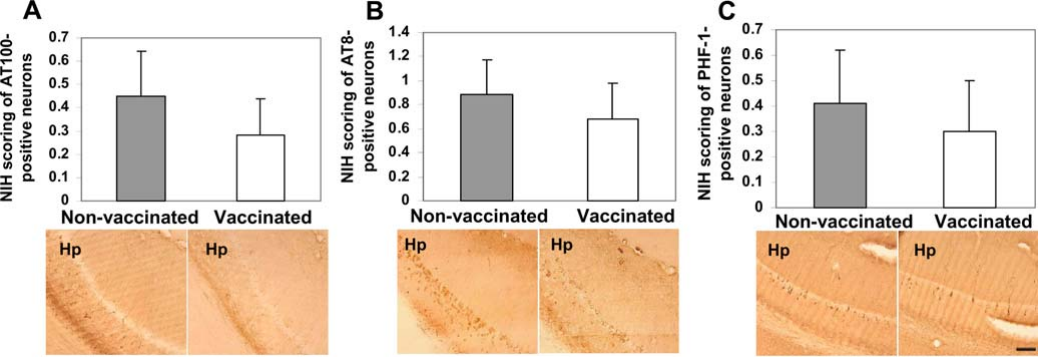
Decrease in the levels of phosphorylated tau detected in the brains of vaccinated mice was not statistically significant. A) Image analysis of immunohistochemical staining demonstrates no differences in the levels of early-stage phosphorylated tau (AT100) in the hemibrains of vaccinated and control mice. Representative images of hippocampal regions (Hp) from vaccinated and non-vaccinated mice are presented (scale bar 100 µm). B) Image analysis of immunohistochemical staining demonstrates no differences in the levels of mid-stage phosphorylated tau (AT8) in the hemibrains of vaccinated and control mice Representative images of hippocampal regions (Hp) from vaccinated and non-vaccinated mice are presented (scale bar 100 µm). C) Image analysis of immunohistochemical staining demonstrates no differences in the levels of late-stage phosphorylated tau (PHF) in the hemibrains of vaccinated and control mice Representative images of hippocampal regions (Hp) from vaccinated and non-vaccinated mice are presented (scale bar 100 µm).

Even though Aβ deposition may be the primary event in AD pathogenesis, it is clear that tau pathology also plays an important role in the progression of this disease [37]. Current data suggest that Aβ pathology emerges prior to tau pathology and may accelerate neurofibrillary tangle (NFT) formation [38], [39]. More recently, it was shown that early-, but not mid- and late-stage tau pathology could be significantly reduced in very old 3xTg-AD mice following active or passive vaccination [40]. We did not observe changes in the number of HT-7 positive neurons indicating that vaccination did not decrease total tau accumulation (Figure 7A). Likewise, we did not observe a significant reduction in early-(AT100), mid-(AT8), and late-(PHF-1) levels of hyperphosphorylated tau after DNA vaccination (Figure 8). To confirm this data, we also used more sensitive quantitative ELISA method and demonstrated that the levels of soluble tau were unaffected by DNA vaccination (Figure 7B). Although, concentration of insoluble tau decreased in brains of vaccinated animals compared with that of control mice, this change was not significant mainly due to a large variability of tau pathology in individual animals (Figure 7C). Thus, we concluded that even though tau burden decreased in immune 3xTg-AD mice, this change was not significant.

### DNA epitope vaccine decreases inflammation and does not induce hemorrhages in immune mice

**Figure 9:**
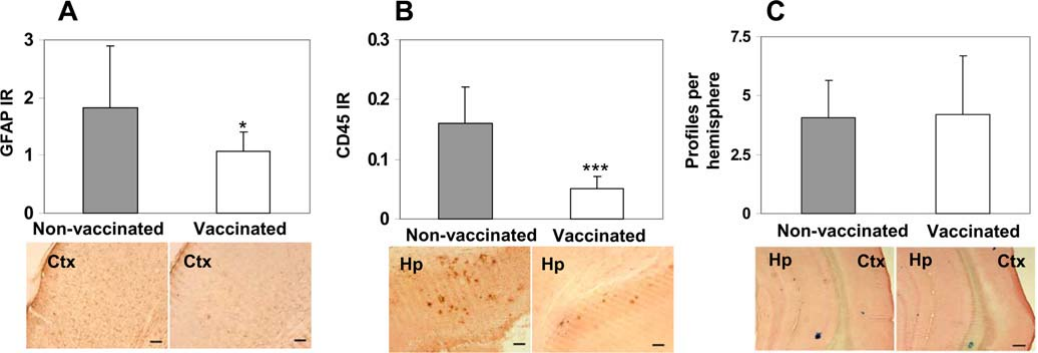
Vaccination with DNA epitope vaccine leads to less astrocytosis and microglia activation without inducing cerebral hemorrhages. A) Image analysis of hemibrains stained with anti-GFAP antibody showed significantly less astrocytosis (P<0.05) in mice vaccinated with DNA epitope vaccine in comparison with control mice (scale bar 50 µm). Representative images of cortical regions (Ctx) from vaccinated and non-vaccinated mice are presented. B) Image analysis of hemibrains from immunized or control mice, stained with anti-CD45 antibody, showed significantly less microglia activation (P<0.001) in mice immunized with DNA epitope vaccine. Representative images of hippocampal regions (Hp) are presented (scale bar 50 µm). C) Level of microhemorrhages in the hemibrains of vaccinated mice did not differ from that in control animals. Image analysis of hemibrains from vaccinated or control mice were performed after Prussian blue staining. Of note, characteristic blue hemosiderin-positive profiles were observed in the neocortical, leptomeningeal, hippocampal and thalamic areas of hemibrain. Representative images of cortical (Ctx) and hippocampal (Hp) regions are presented (scale bar 200 µm).

To examine inflammation-related pathology, the same brain regions of immunized and control 3xTg-AD mice used for Aβ burden studies were evaluated for glial activation, lymphocyte infiltration and microhemorrhages in blood vessels. A quantitative image analysis of brain tissues stained with anti-GFAP and anti-CD45 antibodies indicated that reduction of amyloid plaques in the brains of immune 3xTg-AD mice led to less astrocytosis (P<0.05) and microglial activation (P<0.001), respectively (Figure 9A, B).

Previously, it was reported that passive transfer of anti-Aβ antibody [41] or active immunizations with Aβ_42_ formulated in strong adjuvants, CFA/IFA [42] may induce cerebral microhemorrhages in ∼20 months old APP/Tg mice. However, microhemorrhages have not been reported in aged 3xTg-AD mice. Characteristic blue hemosiderin-positive profiles were observed primarily in the neocortical, leptomeningeal, hippocampal and thalamic areas of the brain at a rate of 4.08±1.59 and 3±2.04 per hemibrain in control non-immunized and vaccinated mice, respectively (Figure 9C). Thus, the DNA epitope vaccine does not increase the incidence of cerebral microhemorrhages, suggesting its safe and unique nature. In support and in accordance with our previous data [21], [24], no T cells (CD3^+^, CD4^+^, or CD8^+^ positive) were detected in the brains of immunized or control 3xTg-AD mice (data not shown).

## Discussion

Current interest in immunotherapy for AD is based on the amyloid cascade hypothesis [1] and the fact that Aβ specific antibody can inhibit accumulation and facilitate the clearance of Aβ deposits from the brains of various AD mouse models [3]–[5], [7] and AD patients [8]–[15]. Although the first clinical trial with the AN-1792 vaccine was halted because of unexpected cerebral inflammation detected in a subset of participants, presumably due to infiltration of autoreactive T cells rather than anti-Aβ antibody, caused the significant adverse effects in the vaccinated patients [9], [10], [12]. In fact, the presence of high titers of anti-Aβ antibody in the sera of vaccinated mice [21], [43] correlates with the reduction of Aβ pathology and was linked to improved spatial learning for vaccinated PDAPP mice [43]. High titers of antibody may also be therapeutically beneficial in AD patients [13], [14]. Herein, we report that our DNA epitope vaccine induces very robust anti-Aβ humoral and Th2-polarized immune responses, and prevents the development of cognitive deficits in aged 3xTg-AD mice. Importantly, the DNA epitope vaccine does not generate autoimmune cellular responses specific to Aβ similar to that reported with a peptide epitope vaccine [20], [21]. Therefore, this DNA epitope vaccine may represent a more effective and safer form of active immunotherapy in humans given the induced Th2-type of immune response, less activated microglia and astrocytosis due to the reduction of Aβ plaques, and the lack of immunotherapy-induced microhemorrhages in vaccinated 3xTg-AD mice.

The attractive feature of a vaccine including MDC/CCL22 as the molecular adjuvant is that it is controlled by the expression of Th2-type chemokine that plays a critical role in the antigen-induced recruitment of Th2 cells via chemotaxis, activation of CCR4-expressing Th2-type CD4^+^T cells [44]–[46] followed by B-cell activation [29]. These features of MDC have been associated with its superb efficiency to induce humoral and Th2 responses without detectable CD8^+^ T cell responses when used as fusions with weakly immunogenic antigens [29]. Typically, approaches that target various endocytic cell surface receptors are known to increase the efficiency of antigen presentation between 100- to 10,000-fold [47]. In accordance with our previous reports on the mechanism of chemokine-based vaccines [48], [49], we believe that our DNA epitope vaccine was efficiently delivered and internalized into endo/lyzosomal compartments of target CCR4^+^ Antigen-Presenting Cells (APCs). As a result, Aβ and PADRE were also processed and presented onto MHC class II molecules to maintain a needed Th help for generation of anti-Aβ antibody. In fact, only few micrograms of DNA epitope vaccine were sufficient for induction of strong anti-PADRE Th2-polarized responses and high levels of anti-Aβ antibody in wild-type and 3xTg-AD mice (Figures 2 and 3). One may suggest that molecular adjuvant incorporated into our DNA epitope vaccine could lead to the production of Th2 cytokines that, in turn, may cross the Blood Brain Barrier and contribute to Aβ clearance in the brains of vaccinated 3xTg-AD mice. However, the measurement of Th1 and Th2 cytokines in the brain extracts of experimental and control animals (non-vaccinated and injected with MDC-irrelevant antigen) demonstrated that this is not the case. In fact, immunizations with plasmids, encoding either AD epitope vaccine or irrelevant antigen, did not alter concentrations of cytokines in the brains of mice (data not shown).

The beneficial effects of anti-Aβ antibody in reducing the soluble Aβ forms have been emphasized, because Aβ oligomers appear to play a critical role in the pathogenesis of AD [2]. Our DNA vaccine generated high titers of antibody specific to Aβ inhibiting the accumulation of soluble/oligomeric forms of Aβ peptide (Figure 5) and significantly reduced development of age-related behavioral impairment in 3xTg-AD mice (Figure 4). Another important issue is the association of tau pathology with Aβ accumulation. Data from AD patients and mouse models of AD suggested that Aβ pathology emerges prior to tau pathology and accumulation of Aβ results in increased NFT formation [38], [39]. Previously, it was shown that the number of AT100, but not AT8- and PHF-1-positive neurons and NFT was reduced in ∼24 mo old 3xTg-AD mice following active or passive vaccination [40]. In our experiments, the DNA epitope vaccine did not significantly reduce the number of total tau-positive neurons (Figure 7). We did not observe significant changes in early-, mid-, and late-stages of hyperphosphorylated tau (Figure 8), as well as the levels of soluble and insoluble tau in immune mice (Figure 7). These differences between our data and [40] may be accounted for tau pathology not fully developed in our colony of 18±0.5 mo old 3xTg-AD mice and the difference between immunized and control animals may be detectable at later ages.

In recent years, DNA vaccine technology has received considerable scientific interest because of significant advantages of DNA immunization in comparison to other means, i.e. ease of vaccine development, high stability of the preparation, capability of modifying genes encoding desired antigen/s, ability to target cellular localization of an antigen by means of adding or removing signal sequences or transmembrane domains, and the ability to selectively elicit the desired type of immune response (humoral or cellular and proinflammatory or anti-inflammatory [50].

Using this strategy, we reported for the first time on the efficacy of DNA vaccine based on Aβ_42_ [24]. Later, several other groups confirmed these data and tested similar types of DNA vaccine in wild-type and APP/Tg mice [25]–[28], [51]. However, in all these studies [25]–[28], [51] and in our previous experiments [17] only low titers of anti-Aβ antibody were generated in vaccinated APP/Tg mice. It is undesirable, because it is known that DNA vaccines are even less effective in large animals and humans [50]. A DNA vaccine based on a full-length Aβ_42_ would not, in theory, be an improvement to the AN-1792 peptide vaccine because it contains the self-Th cell epitopes [52], [53]. Of note, the recent claim of Ocura and colleagues [51] that Aβ_42_-based DNA vaccine induced low titer of antibody without generation of autoreactive Th responses is unsubstantiated, because Aβ_42_ peptide is the T cell-dependent antigen and requires help from CD4^+^T cells (data not shown). In sharp contrast with these studies, our DNA epitope vaccine did not have Aβ Th-cell epitope and, therefore, could not generate autoreactive Th cells, while inducing strong non-self responses to foreign T cell epitope that was incorporated in this immunogen.

In summary, we have developed an efficient and simple DNA-based AD vaccine strategy that uses the self-B cell epitope from Aβ, a non-self promiscuous T cell epitope, and a strong molecular adjuvant, MDC. This DNA epitope vaccine is likely to be effective and safe in humans, because it (i) should induce strong antibody responses to Aβ without generation of autoreactive Th cells, (ii) uses PADRE, a promiscuous synthetic Th epitope that is known to be very effective in the general human population; (iii) uses human MDC that will activate anti-inflammatory Th2-type cells specific to the foreign antigen that is not expressed in human brain. In addition, our preclinical data demonstrate that the DNA epitope vaccine can also significantly decrease microglial activation and astrocytosis without increasing the incidence of microhemorrhages. Future safety and immunology studies in large animals with the goal toward achieving effective humoral immunity and the lowest rate of adverse events should help to translate our DNA epitope vaccine to human clinical trials.

## Methods

### Mice

All animal research protocols were approved by the UCI Institutional Animal Care and Use Committee. 3xTg-AD mouse model develops two age-related neuropathological features associated with AD, amyloid plaques and neurofibrillary tangle formation, as well as age-related behavioral deficits that correlate with the neuropathology. Three to four month old 3xTg-AD homozygous mice (provided by the UCI-Alzheimer's disease research center (NIH/NIA P50 AG16573) and C57/Bl6 (Jackson Lab, MN) were housed in a temperature- and light-cycle controlled animal facility at the Institute for Brain Aging and Dementia (IBAD), University of California Irvine (UCI). Animal use protocols were approved by the Institutional Animal Care and Use Committee of UCI and were in accordance with guidelines of the National Institutes of Health.

### DNA Constructs

3Aβ_1–11_-PADRE minigene was generated using overlapping PCR technique. Each copy of Aβ_1–11_ was separated from the other by GS small linker. As a mammalian expression plasmid we used pCMVE-AB/MDC expressing MDC fused in frame with IP10 signal sequence [29]. 3Aβ_1–11_-PADRE minigene was cloned into pCMVE-AB/MDC in frame to the 3′-end of MDC gene using XhoI/BamHI restriction sites. pCMVE-AB vector contains CMV promoter/enhancer and an ampicillin-resistance gene. 3Aβ_1–11_-PADRE and MDC genes were fused through spacer sequence Asn-Asp-Ala-Gln-Ala-Pro-Lys-Ser. The correct sequence of the generated plasmid was confirmed by nucleotide sequence analysis. Plasmids were purified using Endofree plasmid purification kit (Qiagen, CA) as recommended by manufacturer. The expression of the plasmid was detected in transfected CHO cells by immunoprecipitation/western blot or flow cytometry as previously described [24]. Both IP and WB were performed with anti-Aβ 6E10 monoclonal antibody (Signet, MA). In addition, WB with anti-human CCL22/MDC antibody (R&D systems, MN) was also performed. Proteins were visualized with enhanced chemiluminescence detection using Luminol reagent (Santa Cruz Biotechnology, CA). Expression of pMDC-3Aβ_1–11_-PADRE in transfected CHO cells was analyzed using Flow cytometry assay. Briefly, the transfected CHO cells were fixed and permeabilized by incubation (20 min at 4°C) with Perm/Fix Buffer (BD Pharmingen, CA). After washing, cells were stained with primary 6E10 anti-Aβ antibody (Signet, MA) for 30 min at 4°C, and then with FITC-conjugated rat anti-mouse secondary antibody (BD Pharmingen, CA) 30 min at 4°C. Samples were analyzed using FACScan and CellQuest software (BD Biosciences, CA).

### Immunization

Three groups of 3xTg-AD mice were included in these studies: pMDC-3Aβ_1–11_-PADRE (n = 13), pMDC-irrelevant control (n = 5) and a group of untreated animals (n = 5). The experimental plasmid was administered by gene gun bombardment (Bio-Rad, CA) as recommended by manufacturer and previously described [24]. Each bullet carried 0.5 mg gold (Bio-Rad, CA) coated with 3 µg DNA. Each mouse received 3 shots (total 9 µg DNA) on shaved abdominal skin using the gene gun with a helium gas of 400 psi. Three vaccinations were performed with a 2-week interval and continued with one and half monthly immunizations (total 11). Four mice from the vaccinated group were sacrificed on 7^th^ day after the fifth immunization, and cellular immune responses were analyzed.

### Detection of anti-Aβ antibody in serum and their functionality testing

On day 7 after each immunization blood was collected for analysis of anti-Aβ antibody by ELISA as we described earlier [20], [21], [23], [24]. The reaction was developed by adding 3,3′,5,5′tetramethylbenzidine (TMB) (Pierce, IL) substrate solution and stopped with 2M H_2_SO_4_. The optical density (OD) was read at 450 nm (Biotek, Synergy HT, VT), and antibody concentrations were calculated using a calibration curve generated with 6E10 monoclonal antibody (Signet, MA). HRP-conjugated anti-IgG1, IgG2a^b^, IgG2b and IgM (Zymed, CA) antibody were used for characterization of isotype profiles. Dissociation of anti-Aβ antibody from Aβ in sera was performed at pH 3.5 as described earlier [33]. Sera from immunized mice were also screened for the ability to bind to Aβ plaques in the human brain as we previously described, using immunohistochemistry [24]. A digital camera (Olympus, Japan) was used to capture images of the plaques at 20× magnification. The binding of antisera (dilution 1∶1,000) to the β-amyloid plaques was blocked by preabsorption of the sera with 5 µM of Aβ_1–11_ and Aβ_42_ peptides (1 hr, 37°C).

### T cell proliferation

To detect T cell proliferation we used sucinimidyl ester of carboxyfluorescein diacetate (CFSE) assay as we described previously [21]. Briefly, to detect antigen-specific proliferation of CD4^+^ cells, we stained splenocytes cultures with 1 µM CFSE (Molecular Probes, OR) for 10 min at 37°C. After washing, cells were incubated 3 days in AIM-V media alone or with 2.5 µM of PADRE peptide. After incubation, cultures were stained with PE-labeled rat anti-mouse CD4 monoclonal antibody (BD Pharmingen, CA). Since dead cells might fluoresce non-specifically, these cells were excluded from the assay using a nucleic acid dye (7-amino actinomycin D, 7-AAD from BD Pharmingen, CA), and proliferation of viable cells was analyzed by FACScan flow cytometer (BD Biosciences, CA) as described by manufacturer. CD4^+^ T cells population was analyzed using CellQuest software (BD Biosciences, CA).

### Production of cytokines induced by DNA vaccination

Since IL-4 is a critical cytokine associated with Th2 response, while INF-γ is a marker of Th1 response, it is important to analyze the presence of these cytokines. FACS was used to detect the intracellular cytokines by subsets of T cells as previously described [21]. Briefly, cultures of splenocytes from experimental and control animals were restimulated for 3–4 days with PADRE peptide (2.5 µM) and then for 4–6 hr with PMA and ionophore (ionomycin) (both Sigma, MO). In addition, we added brefeldin A (BD Biosciences, CA) to block cytokine secretion, which increases intracellular accumulation. Surface staining was performed using FITC-labeled anti-mouse CD4 and CD8 MoAb (BD Pharmingen, CA). Cells were permeabilized, and production of IL-4 or IFN-γ by CD4^+^ and CD8^+^ T cell subsets was detected using the appropriate PE-labeled anti-mouse antibody (BD Pharmingen, CA). The percentages of CD4^+^ INF-γ^+^, CD4^+^IL-4^+^, and CD8^+^IFN-γ^+^ cells were calculated in populations of CD4^+^ or CD8^+^ T cell subsets using CellQuest software (BD Biosciences).

### Detection of different cytokines concentrations in brain extracts

Five different cytokines (IL-2, IL-4, IL-5, IFN-γ and TNF) were simultaneously detected in brain extracts of experimental and control mice by Flow cytometry using Mouse Th1/Th2 Cytokine CBA kit as recommended by manufacturer (BD Biosciences, CA).

### Behavior testing: Morris Water Maze

Behavioral testing in 3xTg-AD and wild-type (C57/Bl6) mice was performed as described in [36]. Briefly, 18±0.5 mo old mice, that had received 11 vaccinations, were trained to swim in a Morris Water Maze (MWM), an aluminum tank of 1.5 m diameter with white painted walls and filled with opaque water maintained at 25–29°C. MWM was located in a room with visual cues. Prior to training, mice were placed on hidden platform for 10 s to reduce stress. Mice were trained to swim to clear Plexiglas platform submerged 1.5 cm beneath the surface of the water and invisible to the mice while swimming. For each mouse, the location of a platform was chosen randomly but was kept constant through the training. On each trial the mouse was released into the tank at one of four designated start locations and allowed to find and escape onto the platform. If a mouse failed to find the platform within 60 s, it was manually guided to the platform and allowed to remain there for 5 s. After this, each mouse was placed into a holding cage under a warming lamp for 25 s until the start of the next trial. During the learning period each animal was subjected to a daily four-trial session for 12 consecutive days until mouse has reached the criterion (<20 s escape latency). Retention of a spatial training was assessed 1.5 and 24 hrs after the last training trial. Both probe trials consisted of 60 s free swimming in the pool with the platform removed. Mice were monitored with the camera mounted on the ceiling directly over the pool and recorded on video tape for subsequent analysis. The parameters measured during the probe trial were (1) escape latency to cross the platform location, (2) number of platform location crosses, and (3) time spent in the quadrant opposite to the one containing the platform during training.

### Brain collection

Immediately after the final behavioral test, both vaccinated and non-vaccinated mice were sacrificed for further neuropathological analysis. Mice were anesthetized with Nembutal (40 mg/kg) and transcardially perfused with ice-cold PBS. Each mouse's skullcap was surgically removed; brain was quickly excised and split into halves by a single mid-saggital cut. The left hemisphere was snap frozen and reserved for measurement of both soluble/insoluble Aβ_40_ and Aβ_42_ levels by ELISA as well as tau quantification by ELISA. The right hemisphere was fixed in 4% paraformaladehyde in PBS at +4°C for 36 hrs for further sectioning.

### Determination of Aβ and tau levels in soluble/insoluble fractions

To determine the levels of both soluble/insoluble Aβ_40_ and Aβ_42_, frozen left hemispheres of all animals were homogenized (150 mg/mL) in tissue homogenization buffer (2% SDS in TBS) with protease inhibitors (Sigma, MO) and phosphatase inhibitors (Calbiochem, CA). Each sample was briefly sonicated to sheer the DNA, then centrifuged at 100,000×g for one hour at +4°C. Supernatant from each sample was drawn off and stored at −80°C for later quantification of soluble Aβ and tau levels. The remaining pellet was solubilized in 70% formic acid (FA) by sonication and centrifuged at 100,000×*g* for 1 hr at +4°C. Avoiding upper lipid layer, the lower aqueous layer was collected and store at −70°C for later quantification of insoluble Aβ and tau levels. Biosource ELISA kits (Invitrogen, CA) were used for detection of both Aβ_40_ and Aβ_42_ as well as total tau levels in the brain extracts. Protein concentration was determined using BCA Protein Assay Kit (Pierce, IL) and samples were adjusted to the equal concentration.

Western Blot with soluble fractions of brain homogenates from immune, control and wild-type mice was performed to detect the level of Aβ oligomers as described in[21]. Briefly, proteins were immunoprecipitated from the brain homogenates of individual mice with anti-Aβ 20.1 monoclonal antibody (Signet, MA), subjected to electrophoresis on to 4-12% Bis-Tris polyacrylamide gel in MES buffer under reducing conditions (Invitrogen, CA) and electrotransferred onto nitrocellulose membrane (GE Healthcare, NJ). The membrane was blocked with 5% fat-free dry milk following by detection of Aβ oligomers using biotinylated 20.1 monoclonal antibody and HRP-streptavidine. Proteins were visualized with enhanced chemiluminescence detection using Luminol reagent (Santa Cruz Biotechnology, CA). Western blots were scanned and converted into digital files to calculate the optical density of Aβ oligomer spots with NIH Image J software, version 1.36b. The relative optical density is presented as average value±SD for each group.

### Immunohistochemistry

Free-floating 50 µm-thick coronal sections of fixed hemibrains were cut using a vibratome, and 6 equally spaced sections from each brain were used in each staining experiment. Aβ deposits were detected with 6E10 (1∶1,000, Signet, MA), activated microglia and astrocytes were stained with the anti-CD45 (1∶300, Serotec, NC) and anti-GFAP (glial fibriliary acidic protein, 1∶2,000, Dako, Denmark) antibody, respectively as we described [21]. To detect the general tau pathology, we used HT7, recognizing epitopes 159–163 (1∶500, Pierce, IL). Phosphorylated tau was detected with AT100 (p-T212, S214, 1∶500, Pierce, IL), AT8 (p-S202, T205, 1∶50, Pierce, IL), and PHF-1 (p-S396, S404; 1∶1,000). As a pre-treatment for anti-Aβ immunostaining, we used 90% formic acid for 4 min, and for anti-CD45, 0.03 mg/ml proteinase K for 5 min at RT. All sections were hydrogen peroxide quenched, blocked and incubated with primary antibody overnight at +4°C. Incubations with appropriate biotinylated secondary antibody and ABC for 1 hr were performed followed by color development using DAB (3,3′-diaminobenzidine) substrate kit (both from Vector Labs, CA). Sections were mounted on Vectabond coated slides (Vector Labs, CA), dehydrated and covered using Depex. Thioflavin S was used to visualize fibrillar Aβ. Briefly, mouse brain sections were washed with Tris buffer and stained for 10 min with a solution of 0.5% Thioflavin S in 50% ethanol, followed by 50% ethanol and Tris buffer, then dried and covered using Vectashield (Vector Labs, CA). Prussian blue staining of ferric acid was performed according to the standard protocol. Sections of mouse brains were stained with Prussian blue solution made of equal parts of 5% potassium ferrocianide and 5% hydrochloric acid, for 30 min at RT. After 30 min, sections were washed in deionized water and counterstained with nuclear fast red.

### Quantitative and semi-quantitative image analysis

NIH imaging was used to analyze the area occupied by β-amyloid, glial and Tau reactivity as described previously [21]. Immunostaining was captured using a Sony high-resolution CCD video camera (XC-77) and NIH image 1.59b5 software. For every animal, six images from the superficial layer and six images from the deep layer (525×410 µm each) of the frontal parietal region in the cortex were captured with a 20× objective. For hippocampal areas, similar imaging was performed for CA1, subiculum and dentate gyrus. The threshold for the detection of immunoreactivity was established and then held constant throughout the image analysis. Thioflavin S-positive plaques and Prussian blue-positive profiles were counted by visual inspection of cortical and hippocampal regions of all stained sections while blind to treatment condition, and the average number of Thio-S and hemosiderin deposits, respectively, was calculated per each brain hemisphere by 2 independent observers.

### Statistical Analysis

All statistical parameters (mean, standard deviation (SD), significant difference, etc.) used in immunology, neuropathology and behavioral studies were calculated using Prism 3.03 software (GraphPad Software, Inc., CA). Statistically significant differences were examined using a t-test, or analysis of variance (ANOVA) and Tukey's multiple comparisons post-test (P value less than 0.05 was considered significantly different).

## References

[1] Hardy JA, Higgins GA (1992). Alzheimer's disease: the amyloid cascade hypothesis.. Science.

[2] Haass C, Selkoe DJ (2007). Soluble protein oligomers in neurodegeneration: lessons from the Alzheimer's amyloid beta-peptide.. Nat Rev Mol Cell Biol.

[3] Schenk D, Barbour R, Dunn W, Gordon G, Grajeda H (1999). Immunization with amyloid-beta attenuates Alzheimer-disease-like pathology in the PDAPP mouse [see comments].. Nature.

[4] Janus C, Pearson J, McLaurin J, Mathews PM, Jiang Y (2000). A beta peptide immunization reduces behavioural impairment and plaques in a model of Alzheimer's disease.. Nature.

[5] Morgan D, Diamond DM, Gottschall PE, Ugen KE, Dickey C (2000). A beta peptide vaccination prevents memory loss in an animal model of Alzheimer's disease.. Nature.

[6] Dodart JC, Bales KR, Gannon KS, Greene SJ, DeMattos RB (2002). Immunization reverses memory deficits without reducing brain Abeta burden in Alzheimer's disease model.. Nat Neurosci.

[7] Bard F, Cannon C, Barbour R, Burke RL, Games D (2000). Peripherally administered antibodies against amyloid beta-peptide enter the central nervous system and reduce pathology in a mouse model of Alzheimer disease.. Nat Med.

[8] Hock C, Konietzko U, Streffer JR, Tracy J, Signorell A (2003). Antibodies against beta-Amyloid Slow Cognitive Decline in Alzheimer's Disease.. Neuron.

[9] Ferrer I, Rovira MB, Guerra MLS, Rey MJ, Costa-Jussa F (2004). Neuropathology and pathogenesis of encephalitis following amyloid-beta immunization in Alzheimer's disease.. Brain Pathol.

[10] Nicoll JA, Wilkinson D, Holmes C, Steart P, Markham H (2003). Neuropathology of human Alzheimer disease after immunization with amyloid-beta peptide: a case report.. Nat Med.

[11] Orgogozo JM, Gilman S, Dartigues JM, Laurent B, Puel M (2003). Subacute meningoencephalitis in a subset of patients with AD after Abeta42 immunization.. Neurology.

[12] Masliah E, Hansen L, Adame A, Crews L, Bard F (2005). Abeta vaccination effects on plaque pathology in the absence of encephalitis in Alzheimer disease.. Neurology.

[13] Bayer AJ, Bullock R, Jones RW, Wilkinson D, Paterson KR (2005). Evaluation of the safety and immunogenicity of synthetic Abeta42 (AN1792) in patients with AD.. Neurology.

[14] Patton RL, Kalback WM, Esh CL, Kokjohn TA, Van Vickle GD (2006). Amyloid-beta peptide remnants in AN-1792-immunized Alzheimer's disease patients: a biochemical analysis.. Am J Pathol.

[15] Nicoll JA, Barton E, Boche D, Neal JW, Ferrer I (2006). Abeta species removal after abeta42 immunization.. J Neuropathol Exp Neurol.

[16] Schenk D (2002). Opinion: Amyloid-beta immunotherapy for Alzheimer's disease: the end of the beginning.. Nat Rev Neurosci.

[17] Cribbs DH, Agadjanyan MG (2005). Immunotherapy for Alzheimer's Disease: Potential Problems and Possible Solutions.. Current Immunology Reviews.

[18] Lemere CA, Maier M, Jiang L, Peng Y, Seabrook TJ (2006). Amyloid-beta immunotherapy for the prevention and treatment of Alzheimer disease: lessons from mice, monkeys, and humans.. Rejuvenation Res.

[19] Agadjanyan MG, Ghochikyan A, Petrushina I, Vasilevko V, Movsesyan N (2005). Prototype Alzheimer's disease vaccine using the immunodominant B cell epitope from beta-amyloid and promiscuous T cell epitope pan HLA DR-binding peptide.. J Immunol.

[20] Mamikonyan G, Necula M, Mkrtichyan M, Ghochikyan A, Petrushina I (2007). Anti-Abeta 1–11 antibody binds to different beta-amyloid species, inhibits fibril formation, and disaggregates preformed fibrils, but not the most toxic oligomers.. J Biol Chem.

[21] Petrushina I, Ghochikyan A, Mktrichyan M, Mamikonyan G, Movsesyan N (2007). Alzheimer's Disease Peptide Epitope Vaccine Reduces Insoluble But Not Soluble/Oligomeric A{beta} Species in Amyloid Precursor Protein Transgenic Mice.. J Neurosci.

[22] Alexander J, Sidney J, Southwood S, Ruppert J, Oseroff C (1994). Development of high potency universal DR-restricted helper epitopes by modification of high affinity DR-blocking peptides.. Immunity.

[23] Ghochikyan A, Mkrtichyan M, Petrushina I, Movsesyan N, Karapetyan A (2006). Prototype Alzheimer's disease epitope vaccine induced strong Th2-type anti-Abeta antibody response with Alum to Quil A adjuvant switch.. Vaccine.

[24] Ghochikyan A, Vasilevko V, Petrushina I, Tran M, Sadzikava N (2003). Generation and chracterization of the humoral immune response to DNA immunization with a chimeric β-amyloid-interleukin-4 minigene.. Eur J Immunol.

[25] Qu B, Rosenberg RN, Li L, Boyer PJ, Johnston SA (2004). Gene vaccination to bias the immune response to amyloid-beta peptide as therapy for Alzheimer disease.. Arch Neurol.

[26] Schultz JG, Salzer U, Mohajeri MH, Franke D, Heinrich J (2004). Antibodies from a DNA peptide vaccination decrease the brain amyloid burden in a mouse model of Alzheimer's disease.. J Mol Med.

[27] Kutzler MA, Cao C, Bai Y, Dong H, Choe PY (2006). Mapping of immune responses following wild-type and mutant ABeta42 plasmid or peptide vaccination in different mouse haplotypes and HLA Class II transgenic mice.. Vaccine.

[28] Kim HD, Jin JJ, Maxwell JA, Fukuchi K (2007). Enhancing Th2 immune responses against amyloid protein by a DNA prime-adenovirus boost regimen for Alzheimer's disease.. Immunol Lett.

[29] Biragyn A, Belyakov IM, Chow YH, Dimitrov DS, Berzofsky JA (2002). DNA vaccines encoding human immunodeficiency virus-1 glycoprotein 120 fusions with proinflammatory chemoattractants induce systemic and mucosal immune responses.. Blood.

[30] Biragyn A, Surenhu M, Yang D, Ruffini PA, Haines BA (2001). Mediators of innate immunity that target immature, but not mature, dendritic cells induce antitumor immunity when genetically fused with nonimmunogenic tumor antigens.. J Immunol.

[31] Monsonego A, Maron R, Zota V, Selkoe DJ, Weiner HL (2001). Immune hyporespobnsivness to amyloid b-peptide in amyloid precursor protein transgenic mice: mplications for the pathogenesis and treatment of Alzheimer's disease.. Proc Nat Acad Sci, USA,.

[32] Petrushina I, Tran M, Sadzikava N, Ghochikyan A, Vasilevko V (2003). Importance of IgG2c isotype in the immune response to b-amyloid in APP/Tg mice.. Neurosci Letters.

[33] Li Q, Gordon M, Cao C, Ugen KE, Morgan D (2007). Improvement of a low pH antigen-antibody dissociation procedure for ELISA measurement of circulating anti-Abeta antibodies.. BMC Neurosci.

[34] Pasquali JL, Soulas-Sprauel P, Korganow AS, Martin T (2007). Auto-reactive B cells in transgenic mice.. J Autoimmun.

[35] Snapper CM, Paul WE (1987). Interferon-gamma and B cell stimulatory factor-1 reciprocally regulate Ig isotype production.. Science.

[36] Billings LM, Oddo S, Green KN, McGaugh JL, Laferla FM (2005). Intraneuronal Abeta causes the onset of early Alzheimer's disease-related cognitive deficits in transgenic mice.. Neuron.

[37] Ballatore C, Lee VM, Trojanowski JQ (2007). Tau-mediated neurodegeneration in Alzheimer's disease and related disorders.. Nat Rev Neurosci.

[38] Lewis J, Dickson DW, Lin W-L, Chisholm L, Corral A (2001). Enhanced Neurofibrillary Degeneration in Transgenic Mice Expressing Mutant Tau and APP.. Science.

[39] Oddo S, Caccamo A, Kitazawa M, Tseng BP, LaFerla FM (2003). Amyloid deposition precedes tangle formation in a triple transgenic model of Alzheimer's disease.. Neurobiol Aging.

[40] Oddo S, Vasilevko V, Caccamo A, Kitazawa M, Cribbs DH (2006). Reduction of soluble Abeta and tau, but not soluble Abeta alone, ameliorates cognitive decline in transgenic mice with plaques and tangles.. J Biol Chem.

[41] Pfeifer M, Boncristiano S, Bondolfi L, Stalder A, Deller T (2002). Cerebral hemorrhage after passive anti-Abeta immunotherapy.. Science.

[42] Wilcock DM, Jantzen PT, Li Q, Morgan D, Gordon MN (2007). Amyloid-b vaccination, but not nitro-NSAID treatment, increases vascular amyloid and microhemorrhage while both reduce parenchymal amyloid.. Neuroscience.

[43] Chen G, Chen KS, Kobayashi D, Barbour R, Motter R (2007). Active beta-amyloid immunization restores spatial learning in PDAPP mice displaying very low levels of beta-amyloid.. J Neurosci.

[44] Bonecchi R, Bianchi G, Bordignon PP, D'Ambrosio D, Lang R (1998). Differential expression of chemokine receptors and chemotactic responsiveness of type 1 T helper cells (Th1s) and Th2s.. J Exp Med.

[45] Sallusto F, Lenig D, Mackay CR, Lanzavecchia A (1998). Flexible programs of chemokine receptor expression on human polarized T helper 1 and 2 lymphocytes.. J Exp Med.

[46] Imai T, Nagira M, Takagi S, Kakizaki M, Nishimura M (1999). Selective recruitment of CCR4-bearing Th2 cells toward antigen-presenting cells by the CC chemokines thymus and activation-regulated chemokine and macrophage-derived chemokine.. Int Immunol.

[47] Zaliauskiene L, Kang S, Sparks K, Zinn KR, Schwiebert LM (2002). Enhancement of MHC class II-restricted responses by receptor-mediated uptake of peptide antigens.. J Immunol.

[48] Biragyn A, Ruffini PA, Coscia M, Harvey LK, Neelapu SS (2004). Chemokine receptor-mediated delivery directs self-tumor antigen efficiently into the class II processing pathway in vitro and induces protective immunity in vivo.. Blood.

[49] Schiavo R, Baatar D, Olkhanud P, Indig FE, Restifo N (2006). Chemokine receptor targeting efficiently directs antigens to MHC class I pathways and elicits antigen-specific CD8+ T-cell responses.. Blood.

[50] Berzofsky JA, Ahlers JD, Belyakov IM (2001). Strategies for designing and optimizing new generation vaccines.. Nature Rev Immunol.

[51] Okura Y, Miyakoshi A, Kohyama K, Park IK, Staufenbiel M (2006). Nonviral Abeta DNA vaccine therapy against Alzheimer's disease: long-term effects and safety.. Proc Natl Acad Sci U S A.

[52] Monsonego A, Zota V, Karni A, Krieger JI, Bar-Or A (2003). Increased T cell reactivity to amyloid beta protein in older humans and patients with Alzheimer disease.. J Clin Invest.

[53] Cribbs DH, Ghochikyan A, Tran M, Vasilevko V, Petrushina I (2003). Adjuvant-dependent modulation of Th1 and Th2 responses to immunization with beta-amyloid.. Int Immunol.

